# Discrete or continuous – aging remains aging

**DOI:** 10.3325/cmj.2020.61.193

**Published:** 2020-04

**Authors:** Branimir K. Hackenberger

**Affiliations:** Department of Biology, Josip Juraj Strossmayer University, Osijek, Croatia *hackenberger@biologija.unios.hr*

The question of the inevitability of death is as old as the human reason. Aging is the term closest to death and the process that leads to it. Why do we age? How do we get older? Can we stop or slow aging? There are innumerable ways in which people have tried to answer these and similar questions. For centuries, alchemists tried to find the “elixir of youth.” As we know, the elixir was not found, but the search for it has led to many important discoveries and helped to establish modern science.

Today, in the 21st century, the learned people are once again engaged in finding answers to the questions of aging. As a result of the research and technological development, human life span has increased significantly over the last two hundred years. In some countries, the average life expectancy is well over 80 years. However, longer life span also brings challenges such as working life extension, predominance of geriatric diseases, and lack of caregivers. The aging of molecular structures, cells, and tissues, the aging of organ systems, and the aging of whole organisms, are still unstoppable processes, although we know more and more about them every day. We even know how to slow them down, but we do not know how to stop them completely.

The sequence from molecular structures to an organism does not end there but also includes the population. Average population age (APA) is the mean age of individuals in a population. According to the age structure of population, we can distinguish between populations in expansion or young populations, stable populations, and old or declining populations. In Europe, no country currently has an expanding population. Italy and Greece have stable populations, while Germany, Bulgaria, and Russia have declining populations. Young populations, ie, populations in expansion, can be found in Nigeria, Saudi Arabia, and Guatemala (1).

Predicting the age structure of the population is of paramount importance to the medical profession. The age structure determines how a whole range of tasks is performed: procurement of specific medicines, the planning and introduction of various types of care, and the professional structuring of medical institutions. Therefore, a good prediction of changes in population age structures is an indispensable part of the development of many strategies. How is it possible to model and predict changes in the age structure of populations?

**Figure 1:**
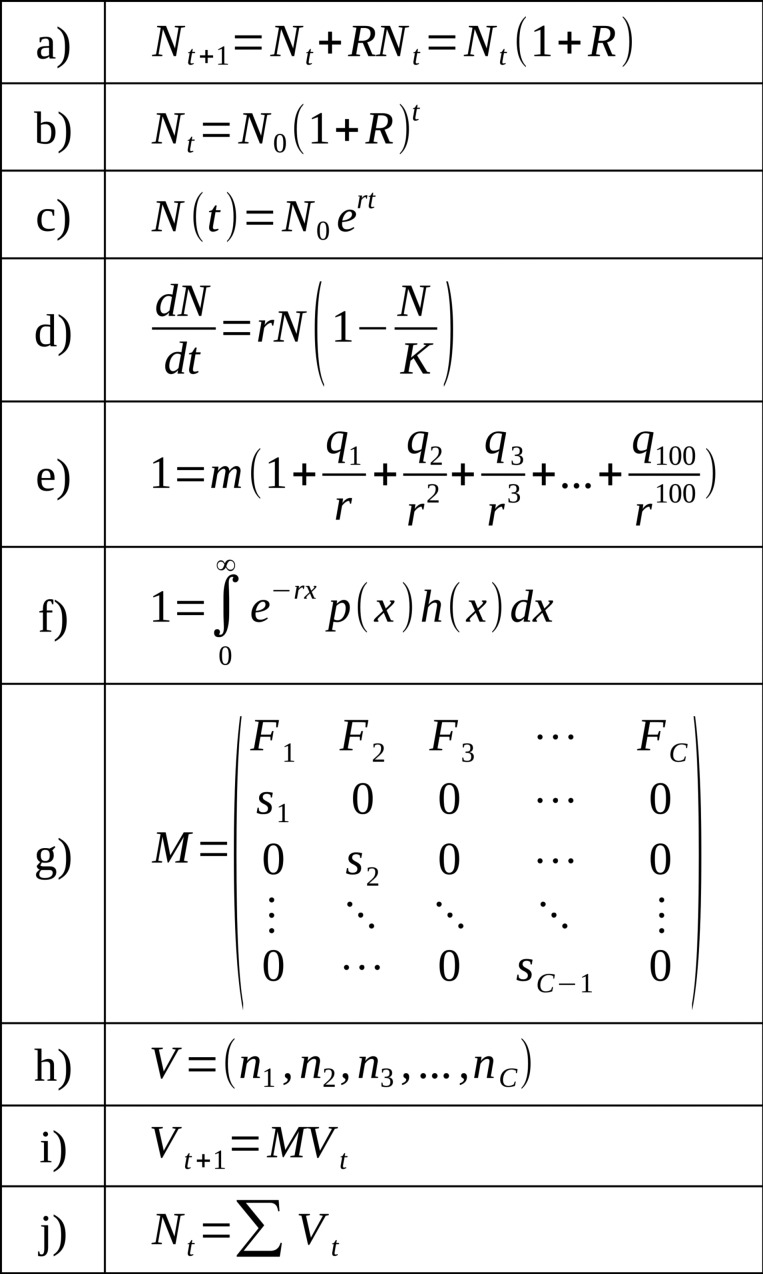
Basic population dynamics equations.

The mathematical description of population growth has attracted many scholars since ancient times (2). In his groundbreaking work, *Introductio in analysin infinitorum* (Introduction to the Analysis of the Infinite), Leonhard Euler presented six examples related to the exponential and logarithmic functions, of which as many as four are concerned with population growth models. Euler assumed that the future population size was equal to the initial population size plus the number proportional to the initial population size (Figure 1a). Furthermore, Euler devised an expression for calculating population size after any period if we know the proportionality factor and the initial population size (Figure 1b). These terms represent the so-called discrete unlimited population growth. A further significant contribution to the theoretical study of population dynamics was made by Thomas Robert Malthus, although the originality of his ideas was disputed. In *An Essay on the Principle of Population*, he outlined a number of problems associated with the growth of human populations. His fear of uncontrolled growth inspired some ideas that today might be considered horrifying. Namely, he proposed building of very narrow streets in poor neighborhoods, which would lead to a faster spread of infectious diseases, with the ultimate purpose of reducing the number of the poor. Although some (2) believe that Malthus plagiarized Euler and other authors, the population growth model described by the expression (Figure 1c) is called the Malthus model of population growth or continuous unlimited population growth model. In this expression, *N_0_* is the initial state of the population and *r* is the so-called Malthusian parameter or, in simpler terms, the coefficient of continuous population growth. It was quickly realized that the growth of animal populations was conditioned by different environmental factors, ie, that it sooner or later became limited. Malthusian population growth was further complemented by Verhulst, who, in his work *Note on the Law of Population Growth*, introduced a new variable *K*, ie, carrying capacity (Figure 1d).

Technological development and the human ability to overcome the natural factors that regulate the population size allowed an indefinite growth of the human population. The growth is sometimes slowed down by world wars or epidemics, but these dangers are surmounted in a relatively short period, whether by scientific advances, technology, or reason. The human population, at least for now, does not know the problem of the *K* variable on the global level.

Euler was also concerned with population growth considering its age structure. He divided the population into age classes and used life tables to calculate the probability of reaching a particular age class. The life tables used by Euler were created by Edmond Halley almost a century before. Even today, similar life tables are produced for individual periods and countries (3). In the book *A General Investigation into the Mortality and Multiplication of Human Species,* he published the term (Figure 1e), which is today called the Euler equation. In this expression, *q_a_* is the proportion of the population that has reached the age of *a*, ie, the survival coefficients, while *m* is the fertility coefficient. Another scientist who dealt with population growth, Alfred Jamel Lotka, was not only interested in the total population but also in the age structure of the population. Unlike Euler, who considered population growth in discrete time periods, Lotka assumed that time was a continuous variable and developed an expression analogous to Euler's that dealt with continuous growth (Figure 1f), in which *p(x)* is the probability of survival to the age *x*, *h(x)* fertility at age *x*, while *r* is the population growth coefficient. This term is called Lotka's equation.

A special contribution to modeling the dynamics of structured populations was made by Patrick Holt Leslie's article *On the Use of Matrices in Certain Population Mathematics*, published in 1945 (4). Specifically, Leslie claimed that by using a structured matrix, the so-called population matrix, it was relatively simple to simulate, besides population size, the changes in the age structure of populations over time. A population matrix is a square matrix with as many rows and columns as there are age classes of the population. The first row of the matrix contains the fertility values for each age class, and the descending subdiagonal contains the probability of survival, ie, reaching the next age class (Figure 1g). To obtain the population structure in the next time step, the matrix is multiplied by vector *V,* representing the population structure in the previous time step (Figure 1h and Figure 1i). The population number at each time *t* is equal to the sum of the members of the vector *V* (Figure 1j).

Owing to the complexity of calculating discrete models, Leslie's model has almost fallen into oblivion. However, with the development of information technology, this way of modeling has returned. There are several reasons for this. First, discrete models are very often more realistic, since the phenomena they describe are often of a discrete nature. Into discrete models of population dynamics, it is relatively easy to enter additional variables that can subsequently be modeled as closely as possible to the real (measured) data. In addition, discrete models can also contain stochastic variables and be used for experiments *in silico*. The results obtained by Leslie's matrix model are often easier to interpret in biological, demographic, epidemiological, or biomedical terms. In addition to changing the population age structure, this method can be used to model almost all discrete phenomena but also the phenomena that can be discretized for modeling purposes.

**Figure 2:**
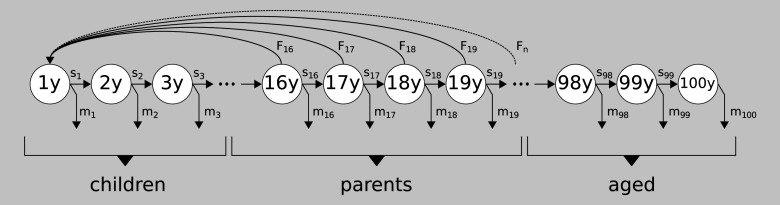
Age structured model of hypothetical human population.

To demonstrate the ease of application of the Leslie's model in predicting age structure, we used this method to investigate how the number of children in a population affects the age structure, that is, the population age over a hundred years. For this purpose, we used a hypothetical structure of the initial population and the life table characteristics of most European countries. The population is divided into 100 age classes assuming that 100 years is the highest possible age (Figure 2).

**Figure 3:**
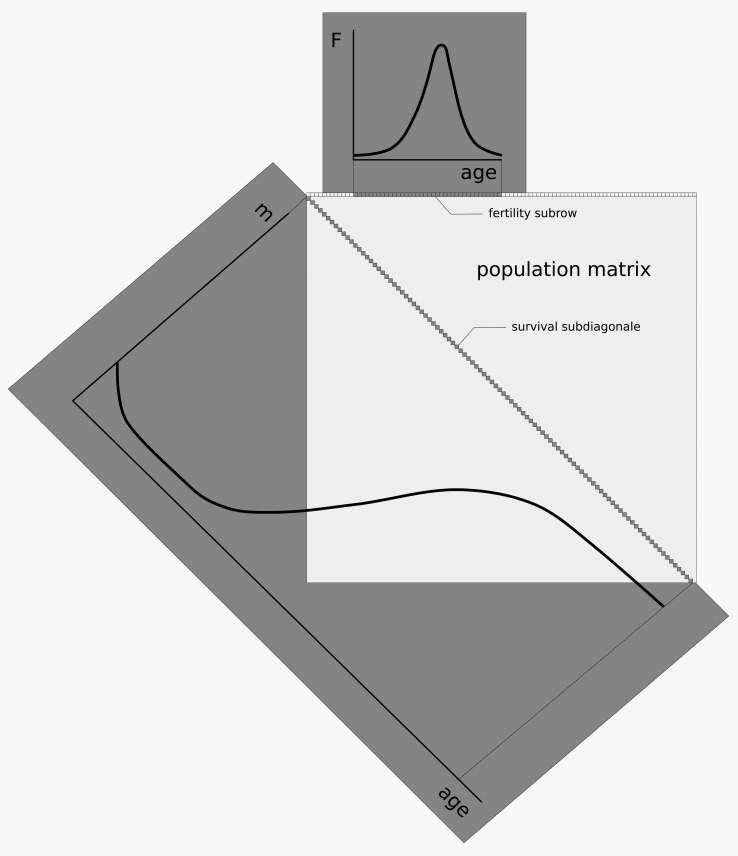
Scheme of population matrix used for simulation.

**Figure 4:**
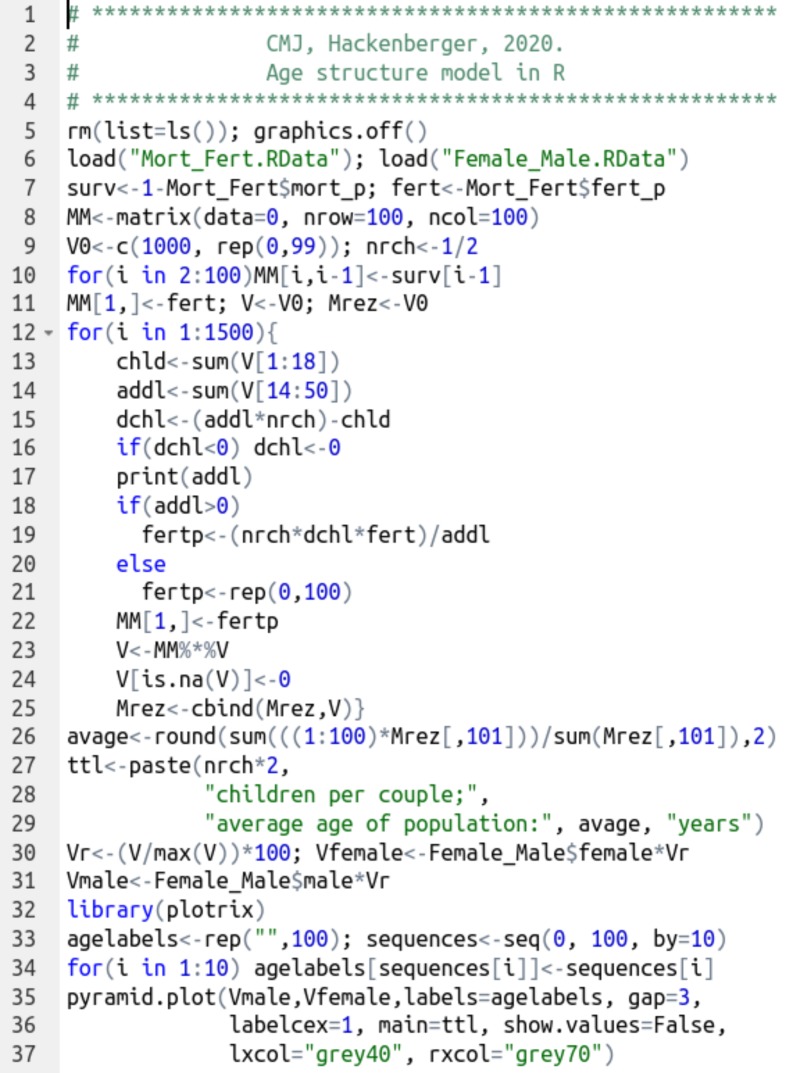
R script for simple age-structured model used for simulation of the long-term (100 years) effects of the average number of children on the age structure of the population and average population age.

**Figure 5:**
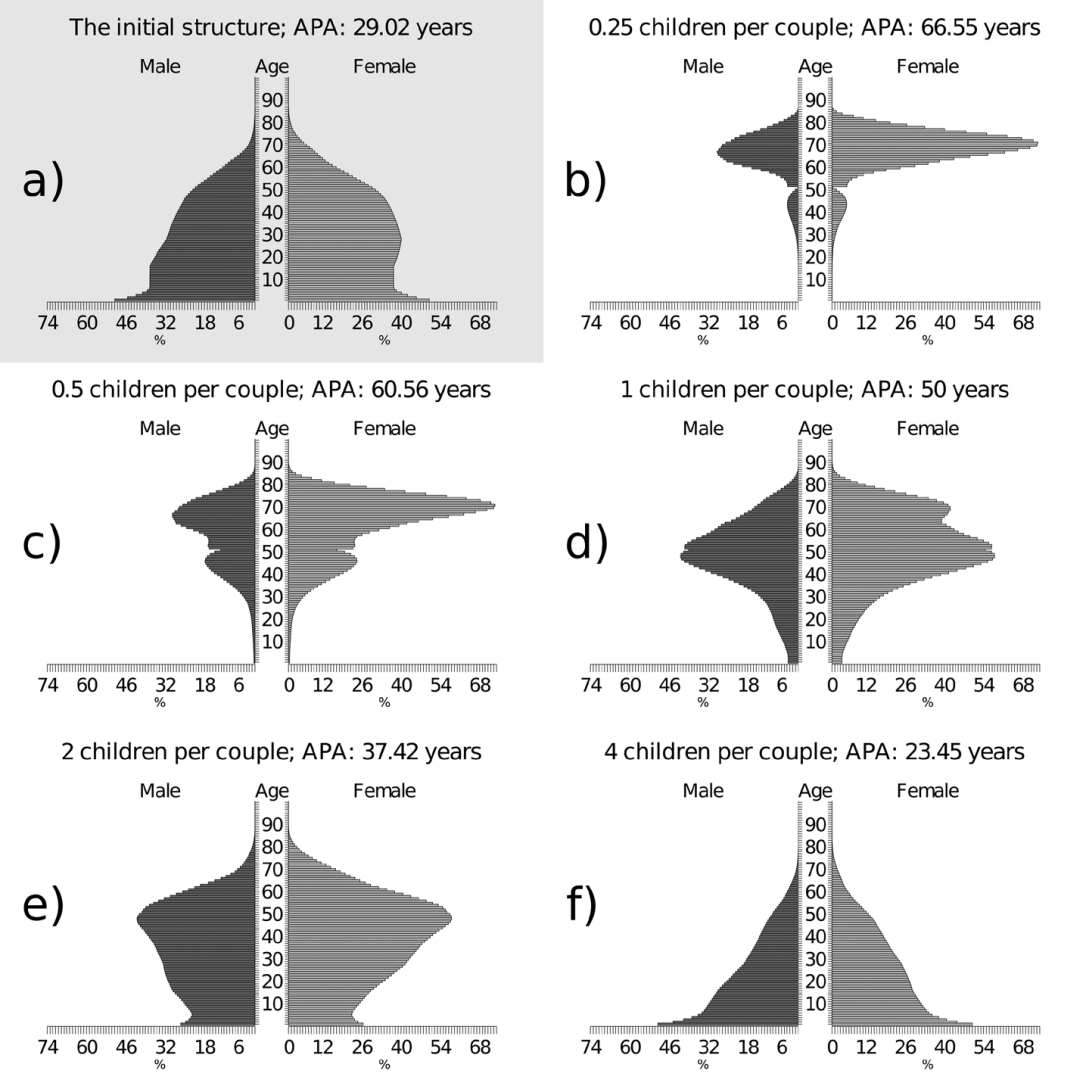
Population pyramids for the initial population and populations with varying average number of children per couple (**A**) initial population; (**B**) 0.25 children per couple; (**C**) 0.5 children per couple; (**D**) 1 child per couple; (**E**) 2 children per couple; (**F**) 4 children per couple). APA stands for average population age.

The following fertility coefficients were assigned to ages 14 to 50 to simulate an average number of children per couple: 0.25 (one child per four couples), 0.5 (one child per two couples), 1 (one child per couple), 2 (two children per couple), and 4 (four children per couple) (Figure 3). The simulation is written in R statistical software environment (5). The simulation code is shown in Figure 4. The simulation results of all five scenarios with respect to the number of children are shown in Figure 5. The results demonstrate how the average number of children in the population influences the change in age structure.

The initial stable population has a trend toward expansion and the mean age of 29.02 years. If over a hundred years, the average number of children does not exceed one in four pairs, that is, if only one out of four couples have a child, the population starts to decline, with a trend toward extinction and with the population age of 66.55 years (Figure 5B). A similar result is obtained with one child per two couples and with only one child per couple (Figure 5C and 5D). If the average number of children is four per couple, the initial population becomes young, ie, expansive population, with a substantially lower average age (23.45 years) in comparison with the initial population (Figure 5F).

Although the results obtained in this way are intuitive and consistent with the experiential data, this is still a simplified hypothetical model. If this approach is to be used to develop predictive simulations of population dynamics and to model the effects of stressors on population structure, it will require a far more rigorous critical approach. In 2019, Kendall et al published a critical review of Leslie's model, with a particular focus on the three biggest drawbacks in terms of possible errors: 1) incorrect fertility parameterization, 2) incorrect estimation of the beginning of reproduction, and 3) under- or overestimation of the growth rate (6). However, most of the conclusions and errors described in this article are related to growth-restricted models and group age categories containing individuals of different ages or with life-stage categories. Such models, eg, Leslie-Lefkovitch models with insect life stages, are far more complex and error-prone. Nevertheless, many models provide highly realistic predictions, especially if the life history of the species whose population is modeled is well known. Demographic models that rest on a modified Leslie model are far simpler. Continuous models seem far more “mathematical” than discrete models, especially if they are full of elegant analytical solutions. However, the most important thing is that the predictive model produces reliable results. Regardless of the model used for predicting the population age structure, if the molecular structures and cells are aging, population will also continue to age. Aging remains aging. The population, in the long run, can only be rejuvenated by having children.
